# ipRGCs Sensitive Blue Light Exposure Promotes the Robustness of Circadian and Neural Stem Cells in Sleep Deprived Conditions

**DOI:** 10.1155/sci/8828183

**Published:** 2025-07-24

**Authors:** Zhaoting Bu, Xiaotong Li, Jinyu Shi, Qiaozhen Qin, Heyang Zhang, Yuanrong Qiu, Lingyu Zhang, Yifei Tan, Hanping Shi, Xiaoxia Jiang

**Affiliations:** ^1^Department of Neuroimmunology and Antibody Engineering, Beijing Institute of Basic Medical Sciences, Beijing 100850, China; ^2^Department of Gastrointestinal Surgery/Department of Clinical Nutrition, Beijing Shijitan Hospital, Capital Medical University, Beijing 100038, China; ^3^Beijing International Science and Technology Cooperation Base for Cancer Metabolism and Nutrition, Beijing 100038, China; ^4^Department of Medical Sciences, Jishou University, Jishou 416000, Hunan, China; ^5^Department of Life Sciences, Anhui Medical University, Hefei 230032, Anhui, China

**Keywords:** blue light, circadian rhythm, neural stem cells, sleep deprivation

## Abstract

Circadian rhythm abnormalities due to sleep deprivation (SD) may promote the development of emotional and cognitive disorders. Though light therapies have been employed to treat circadian disorders, the exact treatments and their underlying biology are still unclear. Our study aimed to investigate the effects of intrinsically photosensitive retinal ganglion cells (ipRGCs) sensitive 480 nm blue light on circadian rhythms affecting emotional and cognitive behaviors and the expression of neural stem cells (NSCs) stemness genes. In this study, we demonstrate that for mice with acute SD for 24 h, exposure to ipRGCs sensitive 480 nm blue light at ~ 1300 lux for 30 min at 8:00 a.m. and 8:00 p.m. improves the stability of disrupted clock genes, increases nocturnal activity, reduces anxiety-like behaviors, and enhances cognitive abilities. Furthermore, 480 nm blue light exposure reduces fluctuations in NSCs stemness gene expression induced by SD, potentially through its effect on enhancing the amplitude of suprachiasmatic nucleus (SCN) circadian oscillations. These findings may provide novel strategy for alleviating rotating circadian rhythm-related anxiety and learning and cognitive obstruction.

## 1. Introduction

Irregular shift work, cross-time-zone travel, and associated sleep disorders are increasingly prevalent in modern society [1, 2]. These disruptions, compounded by exposure to light pollution, can lead to disturbances in sleep and circadian rhythms, which in turn may contribute to metabolic disorders and other diseases [3]. Circadian rhythms are endogenous, ~24-h cycles in physiology and behavior, governed primarily by the suprachiasmatic nucleus (SCN) of the hypothalamus—the master circadian clock in mammals [4, 5]. The SCN synchronizes daily physiological rhythms through neuronal and hormonal signals and maintains coordination with peripheral clocks distributed in organs such as the liver and muscle [6]. Environmental time cues, known as Zeitgebers (ZTs)—with the light–dark (LD) cycle being the most potent—entrain these rhythms by aligning the internal clock to external time [7]. On the molecular level, the circadian system is driven by transcription–translation feedback loops involving core clock genes such as Bmal1, Clock, Per2, and Cry2, which are rhythmically expressed in both the SCN and peripheral tissues [8]. Light is a critical regulator of these clock genes, particularly through a subset of retinal cells known as intrinsically photosensitive retinal ganglion cells (ipRGCs) that detect short-wavelength (blue) light and transmit signals directly to the SCN [9]. Recent studies suggest that neural stem cells (NSCs) are also regulated by circadian rhythms. NSCs in the adult brain, particularly in the subventricular zone (SVZ) and hippocampus, exhibit rhythmic proliferation and differentiation patterns aligned with the circadian system [10–12]. Disruption of these rhythms may impair NSC function, reduce neurogenesis, and contribute to cognitive decline and emotional disturbances [13–15]. Given the wide range of disorders caused by circadian rhythm disruptions, there is an urgent need to develop effective interventions, as current solutions remain limited.

Recent research highlights the distinct effects of different blue light wavelengths on sleep patterns and circadian rhythms [16, 17]. Exposure to short-wavelength blue light (415–455 nm), commonly emitted by electronic devices at night, can disrupt the synchronization between environmental cues and the body's internal clock, often leading to phase delays and impaired sleep quality [18, 19]. In contrast, longer-wavelength blue light at 480 nm has shown promise in promoting cognitive recovery and reducing depressive-like behaviors after traumatic brain injury (TBI) [20]. While previous studies have shown that blue light-filtering lenses can reduce visual fatigue and improve sleep quality [21–23], the specific role of 480 nm blue light in rhythm regulation remains underexplored. Therefore, further investigation of 480 nm blue light is warranted to better understand its impact on circadian modulation.

This study aimed to explore whether 480 nm blue light can restore circadian function disrupted by SD. To test this, we conducted real-time quantitative PCR, immunofluorescence staining, locomotor activity rhythms, and behavioral tests in mice. We observed that 480 nm blue light improved the oscillations of clock genes and altered SCN activation, indicating its potential circadian-resetting effects. Additionally, this light exposure is expected to effectively improve anxiety-like behavior and improve learning and cognitive ability in sleep deprivation (SD) mice, while stabilizing circadian rhythm-related fluctuations in NSCs. The findings of this research may provide new perspectives for understanding the connection between circadian rhythms and health, and play an important role in future treatments of circadian rhythm disorders.

## 2. Materials and Methods

### 2.1. Animals

Eight-week-old C57BL/6J mice were obtained from Shanghai Sipper-BK Laboratory Animal Co. Ltd. (Shanghai, China). The mice were maintained in specific pathogen-free barrier facilities with four animals per cage. Food and water were provided ad libitum. Mice were housed in an environment with a temperature of 20–26°C, relative humidity of 40%–70%, and a 12-h LD cycle (12 h of light from 8:00 to 20:00 with ~200 lux white light intensity). All animal experiments were conducted in accordance with the “Guide for the Care and Use of Laboratory Animals” approved by the Beijing Institute of Basic Medical Sciences. Ethical approval was obtained for all experimental protocols (IACUC-DWZX-2024-530).

### 2.2. SD and Blue Light Exposure

**Figure 1:**
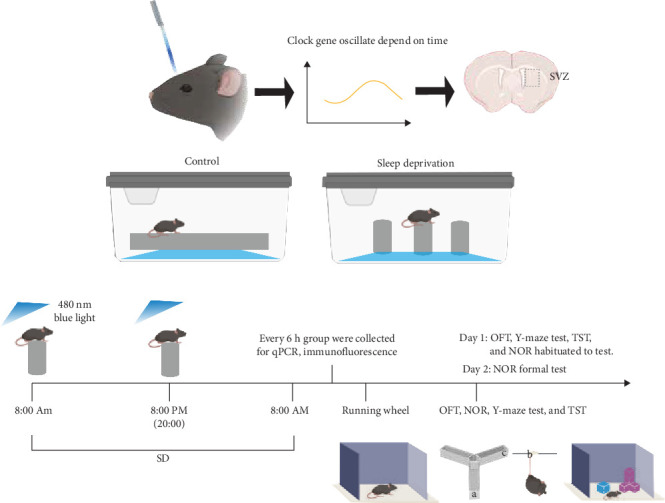
Schematic diagram of the experimental flow.

Mice were placed on large platforms measuring 11 cm in length and width, on small platforms with a diameter of 3 cm (keeping mice in a continuous state of wakefulness), respectively. Both sets of platforms were surrounded by water, and mice in both models had free access to water and food. Acute SD was conducted for 24 h. The duration of SD was based on previous research findings. Blue light treatment was conducted on mice under a 12:12 LD cycle, with ipRGCs sensitive to 480 nm blue light exposure at ~ 1300 lux for 30 min at 8:00 a.m. and 8:00 p.m. (Figure 1). The 1300 lux blue light (~480 nm) used in the experiment had an irradiance of ~7.2 μW/cm^2^ at a distance of 60 cm. Mice were randomly assigned to three groups: control, SD, and SD-blue light. Different sampling time points (ZT0, ZT6, ZT12, ZT18, ZT24) were employed.

### 2.3. Tissue Collection

At different time points, mice were euthanized in batches for perfusion. Mice were anesthetized with aflutin (100 mg/kg, intraperitoneal injection) and perfused intracardially with 0.9% saline followed by 4% paraformaldehyde (PFA). Brains were then extracted and fixed overnight in 4% PFA at 4°C, followed by dehydration in 30% sucrose solution. Coronal brain sections of 30 μm thickness were prepared using a Cryostat microtome (Leica CM3050S) for subsequent experiments.

### 2.4. RNA Extraction and Real-Time Quantitative PCR

Weigh and record the fresh, frozen brain, liver, thymus, and bone marrow tissues separately. Then, add 1 mL of precooled Trizol to each sample. After that, extract and purify total RNA from the samples using chloroform, isopropanol, and 75% ethanol through a series of centrifugation steps. Analyze the RNA concentration and purity using a Nanodrop spectrophotometer. Use a reverse transcription kit (Vazyme) to synthesize cDNA from the RNA. Utilize the cDNA as a template for quantitative PCR (SYBR Green, Vazyme) to determine the expression levels of specific genes. Use the primers (designed and synthesized by Shanghai Sangon Biotech) listed in Table S1 to detect the mRNA expression levels of *Bmal1*, *Clock*, *Cry2*, *Per2*, *Nestin*, and *Sox2* through real-time fluorescence quantitative PCR. Use the Thermo Scientific PikoReal software to analyze the cycle threshold value and calculate the relative mRNA quantity using the 2^–ΔΔCT^ method.

### 2.5. Immunofluorescence Staining

Coronal brain tissue sections were incubated in a blocking buffer (0.01 M PBS, 0.3% Triton X-100, and 3% goat serum) for 1 h in a sealed container. Subsequently, the sections were incubated overnight at 4°C with the following primary antibodies: *cfos* (ab190289, Abcam), *Nestin* (ab6142, Abcam), and *Sox2* (ab97959, Abcam). Following this, the sections were washed three times with PBS for 10 min each, and then incubated at room temperature with Alexa 488 (715-546-150, Jackson Immunoresearch) and Cy3 (711-165-152, Jackson Immunoresearch) for 1 h. To stain the nuclei, Hoechst 33342 (or DAPI) was added to a DAPI solution. The stained sections were analyzed using a fluorescence microscope (Nikon AZ-100 Multifunctional Microscope).

### 2.6. Locomotor Activity Rhythms

SD and SD-blue light group mice were transferred to individual cages equipped with a running wheel (diameter 23 cm, four magnets/wheel) in a ventilated and sound insulation box with chambers with controlled white lighting (200 lux). Wheel-running activity was monitored by an online personal computer connected via a magnetic switch to the running wheel activity system. Wheel revolutions were collected continuously in 24 h.

### 2.7. Behavioral Test

#### 2.7.1. Open Field Test (OFT)

Mice were allowed to adapt to the environment for 1 h, and exploratory and anxiety-like behaviors were measured using an open-field apparatus (50 × 50 × 50 cm) [24]. Each mouse was placed in the center of the open-field apparatus, with the central area defined as a square measuring 10 cm from the walls. The mice movements were recorded using a video tracking system (EthoVisionXT, Netherlands) for 5 min as the mouse was placed in a box (black and opaque on all sides). The box was equipped with a camera and a computer video monitoring system, and the activity of each animal was recorded. The testing apparatus was cleaned with 75% ethanol after each test to prevent residual information and odors from affecting the next animal. The total distance and center zone time was measured to reflect the mouse's autonomous activity ability and anxiety-like symptom.

#### 2.7.2. Tail Suspension Test (TST)

According to previous guidelines [25] a behavioral test for evaluating depressive-like behavior in mice was conducted. Specifically, under normal lighting, a tape was attached to the mouse's tail, and the mouse was hung by the tail from a position at least 15 cm from the single bar (to prevent climbing). The mice's behavior was recorded for 6 min by a camera placed to the side for analysis. The animal was then removed, and the equipment was wiped down with 75% ethanol. Throughout the study, the amount of time the mice were immobile was recorded using a video tracking system, defined as the period when the head or limbs showed no discernible voluntary movement. The first 2 min were excluded due to high stress, and the average immobility time for the last 4 min of each trial was calculated and expressed as seconds. The immobile time was measured to reflect the mouse's depression symptoms.

#### 2.7.3. Y-maze Test

The test equipment consists of a Y-shaped labyrinth composed of three arms (30 cm × 5 cm × 15 cm, named a, b, and c arm). Y-maze test is based on the tendency of the mouse to explore new environments, hence preferring a labyrinth arm not yet explored to an already known one. By the established protocol, a correct alternation is defined as the mouse completing the exploration of the three arms in the sequence a-b-c arm [26]. The experimental sessions have been videotaped by a camera placed above the apparatus.

#### 2.7.4. Novel Object Recognition Test (NOR)

The NOR test is a learning and memory test based on the principle that animals have a natural tendency to explore novel objects. We used an established protocol to assess each mouse with the NOR test [27]. The mice were placed in the box and habituated for 10 min at first day. On the next day, two identical objects (blue cube) were placed in the box, and the mice was allowed to explore the area for 10 min. After 3 h, novel object (irregular cube) and blue cube were placed in the box, and the mice were allowed to explore for 10 min while being recorded by camera. The total time of exploring the novel objects was calculated.

### 2.8. Statistical Analysis

All data were analyzed with Prism 5.0 software (GraphPad Software, San Diego, CA, USA) and are presented as the means ± standard deviations. Statistical significance was assessed by unpaired two-tailed student's tests and one-way ANOVA (*p* < 0.05). Utilize the cosine analysis software circacompare (R package) to obtain the fitted parameters of the cosine curve. The fitted cosine function equation is *Y* = Mesor + Amplitude *⁣*^*∗*^ cos (time_Radians - φ), where Mesor is the baseline/midline. Amplitude is the amplitude of the rhythmic oscillation. Radians is the radian value corresponding to time, and φ is the peak phase.

## 3. Results

### 3.1. 480 nm Blue Light Exposure Maintains Oscillatory Rhythms and Circadian Rhythmicity of Clock Genes

#### 3.1.1. Changes in the mRNA Expression of the Clock Genes

**Figure 2:**
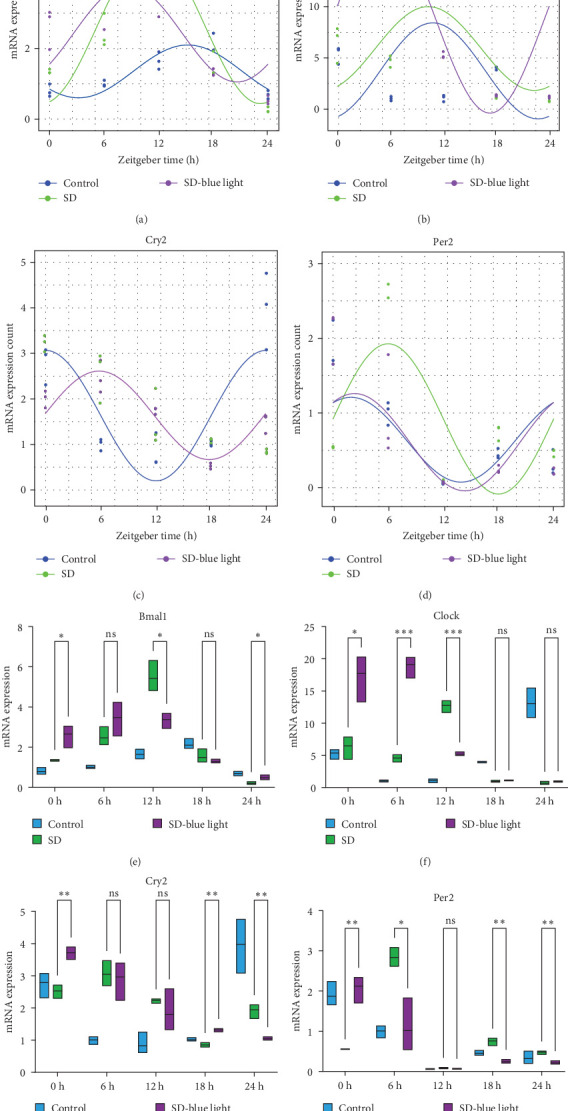
Central clock genes mRNA expression and the rhythmic changes of the fitted cosine function and relative expression changes. mRNA expression of *Bmal1* (a), *Clock* (b), *Cry2* (c), and *Per2* (d), relative expression of *Bmal1* (e), *Clock* (f), *Cry2* (g), and *Per2* (h) (*n* = 8; two-tailed student's tests; *⁣*^*∗*^*p* < 0.05; *⁣*^*∗∗*^*p* < 0.01; *⁣*^*∗∗∗*^*p* < 0.001).

To investigate the effect of blue light exposure on circadian rhythms, we examined the expression of the central clock genes *Bmal1* and *Clock* mRNA, as well as the peripheral clock genes *Cry2* and *Per2* mRNA, in the SCN nuclei and peripheral tissue (liver) using RT-qPCR. Strength of 1300 lux enables rhythm-related genes (*Bmal1*, *Clock*, *Cry2*, *Per2*, etc.) to remain rhythmic. Circadian rhythm disruption is better ameliorated by blue light exposure at 1300 lux, which is the intensity of blue light exposure used in this work (Figure S1). Specifically, 480 nm blue light exposure helps to maintain the circadian rhythmicity of clock genes. In the SCN, the expression of *Bmal1* in the SD-blue light group was phase-advanced compared to both the control and SD groups, with significant upregulation observed at ZT0 and ZT6 (*p* < 0.05). At ZT12, ZT18, and ZT24, no significant differences were observed between the SD-blue light group and the control group (*p* > 0.05, Table S4, Figure 2a,e). A similar trend was found for *Clock* mRNA (Figure 2b), with significant increases at ZT0 and ZT6 in the SD- blue light group (*p* < 0.05, Table S4) compared to the SD group, but no significant differences between the SD- blue light and control groups at other time points (*p* > 0.05). In the SD group, the rhythmic expression of Cry2 was lost, whereas Per2 mRNA expression in the SD-blue light group similar to that of the control group, maintaining its rhythmic pattern (Figure 2c,d,g,h and Table S4). Furthermore, a phase advance was observed. This is reflected in the fact that the peak time hours of the SD- blue light group are earlier than those of the other groups (Table S2).

**Figure 3:**
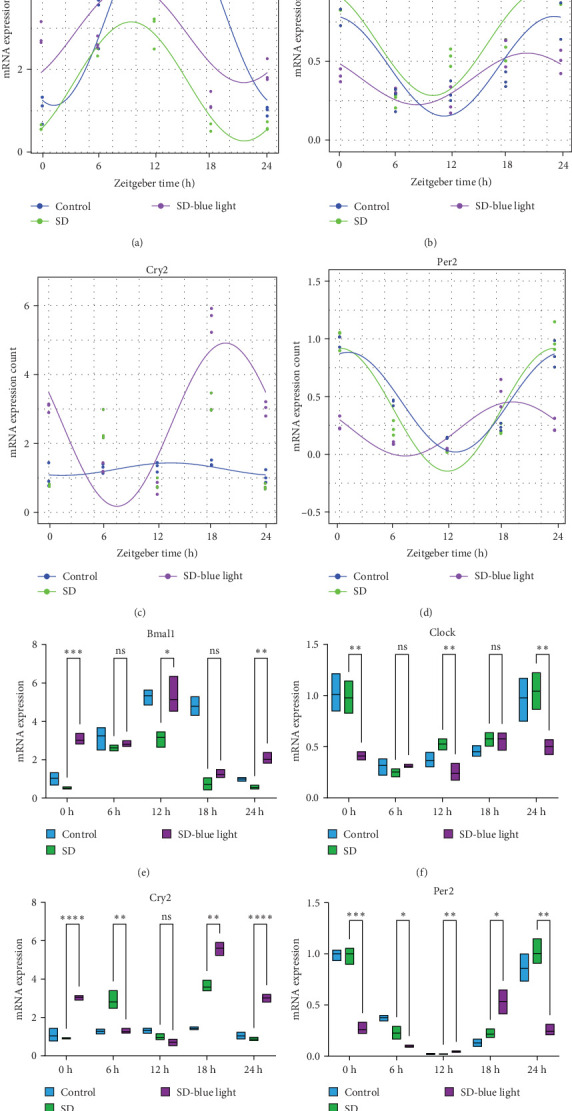
Peripheral (liver) clock genes mRNA expression and the rhythmic changes of the fitted cosine function and relative expression changes. mRNA expression of *Bmal1* (a), *Clock* (b), *Cry2* (c), and *Per2* (d), relative expression of *Bmal1* (e), *Clock* (f), *Cry2* (g), and *Per2* (h).

In peripheral liver tissue, a similar trend was observed for Bmal1 mRNA, with increased expression in the SD- blue light group at ZT0 and ZT18 compared to the SD group (*p* < 0.05, Table S5) (Figure 3a,e). However, Clock mRNA displayed an opposing cosine-like pattern, with the curve opening upwards (Figure 3b,f and Table S5). As in the SCN, the rhythmicity of Cry2 expression was lost in the SD group, while Per2 expression followed a similar trend to Clock in the liver (Figure 3c,d,g,h and Table S5). A phase advance was also observed in the liver tissue. In rhythmic oscillations of Bmall1 and Clock genes, peak time hours were earlier in the SD- blue light group than in the other groups (Table S3).

#### 3.1.2. the Cfos Activation of the SCN Nucleus After Blue Light Exposure

**Figure 4:**
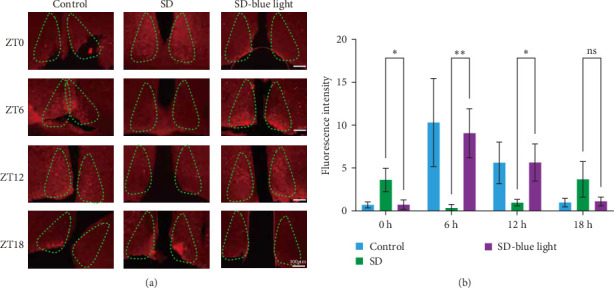
Representative images (a) and quantifications (b) showing activated *cfos* protein of the SCN slice. Scale: 100 μm (two-tailed student's tests; *⁣*^*∗*^*p* < 0.05; *⁣*^*∗∗*^*p* < 0.01).

We next examined the functions of SCN neurons in regulating circadian rhythm by using a cfos activation level. In SD-blue light group, the number of cfos-activated neurons in the SCN decreased at ZT0 and ZT18 (*p* < 0.05), but increase in ZT6 and ZT12, following a pattern similar to the control group. Conversely, the SD group displayed a different *cfos* expression pattern, confirming the impact of SD on SCN function and supporting the beneficial effect of blue light exposure on circadian rhythm (*p* < 0.05) (Figure 4 and Figure S3).

### 3.2. Stabilization of NSCs Stemness Gene Expression Following 480 nm Blue Light Exposure

**Figure 5:**
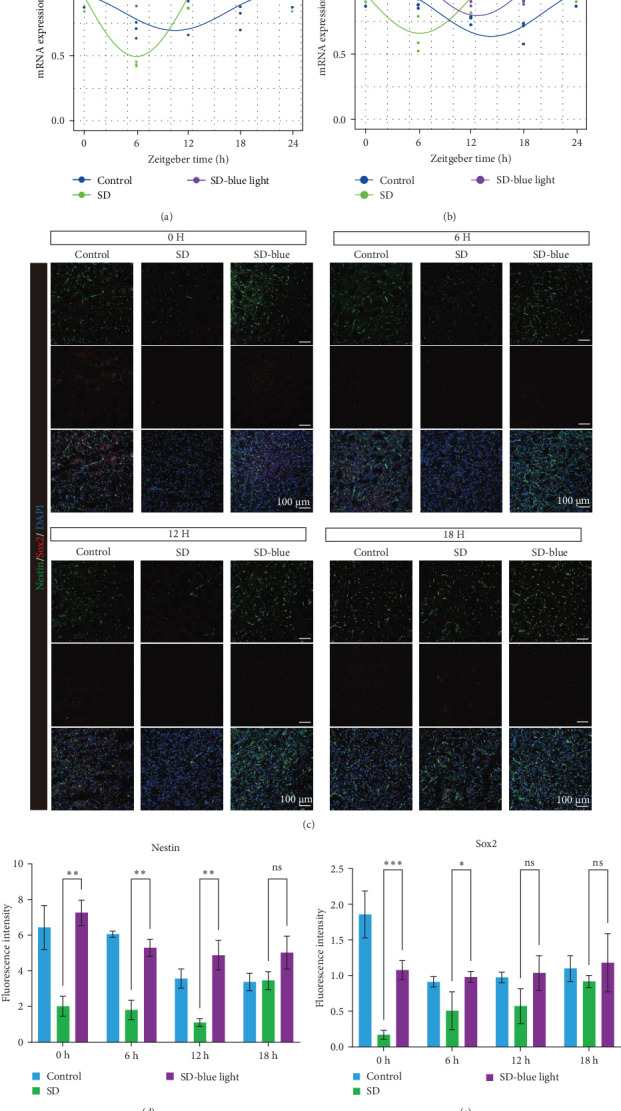
A 480 nm blue light exposure stabilized the expression of NSCs stemness genes. mRNA expression of *Nestin* (a), *Sox2* (b), and the rhythmic changes of the fitted cosine function and relative expression changes. Representative images (c) and quantifications (d, e) showing *Nestin* and *Sox2* expression of the SVZ slice. Scale: 100 μm.

To elucidate the relationship between NSCs, the circadian system, and blue light exposure, we evaluated the expression of NSCs stemness genes. In the SD group, a significant decrease in *Nestin* and *Sox2* mRNA expression was observed at ZT0 and ZT12, while an increase was noted at ZT6 and ZT18 (*p* < 0.05, Table S6), indicating disruption of NSCs stability (Figure 5a,b). Compared to the control group, the number of *Nestin* + NSCs in the SVZ of SD mice was significantly reduced at ZT0, ZT6, and ZT12, whereas no significant changes were observed in the SD-blue light group (Figure 5c). Similarly, the expression of *Sox2* + NSCs showed a similar pattern across the different treatment groups (Figure 5d,e). There were no significant differences in the expression levels or diurnal patterns of *Nestin* and *Sox2* between the 480 nm blue light group and the control group. These findings suggest that exposure to 480 nm blue light stabilizes the fluctuations in NSCs stemness gene expression that are induced by circadian rhythm disruption.

### 3.3. Improved Rhythmic Spontaneous Locomotor Activity in SD-Blue Light Mice

**Figure 6:**
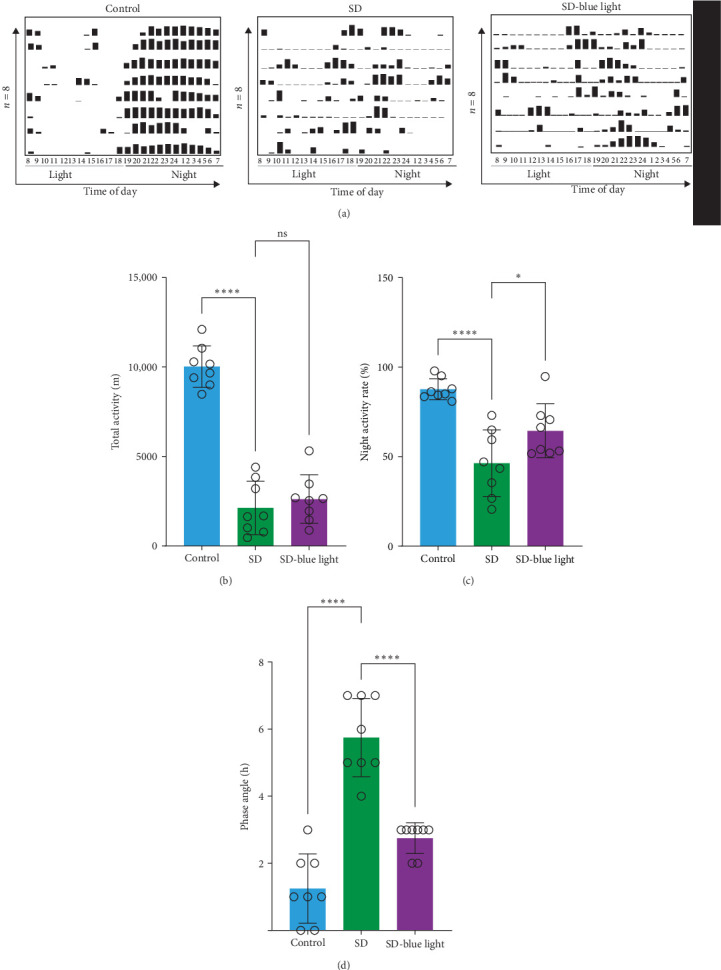
Analysis of locomotor activity and rhythm robustness in a 12 h light–12 h dark (LD 12:12) photoperiod. (a) representative double-plotted actograms of spontaneous locomotor activity of each group mice in LD 12:12 photoperiod. Graphs show the (b) total activity, (c) night activity, and (d) phase angle. A positive phase angle indicates onset of activity prior to lights off; *n* = 8; *⁣*^*∗*^*p* < 0.05, *⁣*^*∗∗*^*p* < 0.01.

Based on the previous findings, we conducted a spontaneous running wheel experiment to further assess verify the circadian rhythmicity after SD. Both the SD group and SD-blue light group exhibited a free-running circadian rhythm of spontaneous locomotor, activity characterized by lower activity during the light phase and higher activity during the dark phase under a light/dark 12:12 cycle (LD 12:12) (Figure 6a). While the total locomotor activity was not significantly different between the SD and SD-blue light groups, both were significantly lower than the control group (Figure 6b and Table S7). Notably, nighttime activity was significantly higher in the SD-blue light group compared to the SD group. Additionally, the onset of activity in the SD-blue light group was closer to the lights-off time, as indicated by a significantly smaller phase angle of entrainment (2.65 ± 0.34 h) compared to the SD group (5.72 ± 1.12 h, *p* < 0.05, Table S7) (Figure 6d). These results indicate that 480 nm blue light exposure improves SD-induced disruptions in rhythmic spontaneous locomotor activity.

### 3.4. 480 nm Blue Light Alleviated Anxiety-Like Behavior and Enhances Improved Learning and Cognitive Abilities in SD Mice

**Figure 7:**
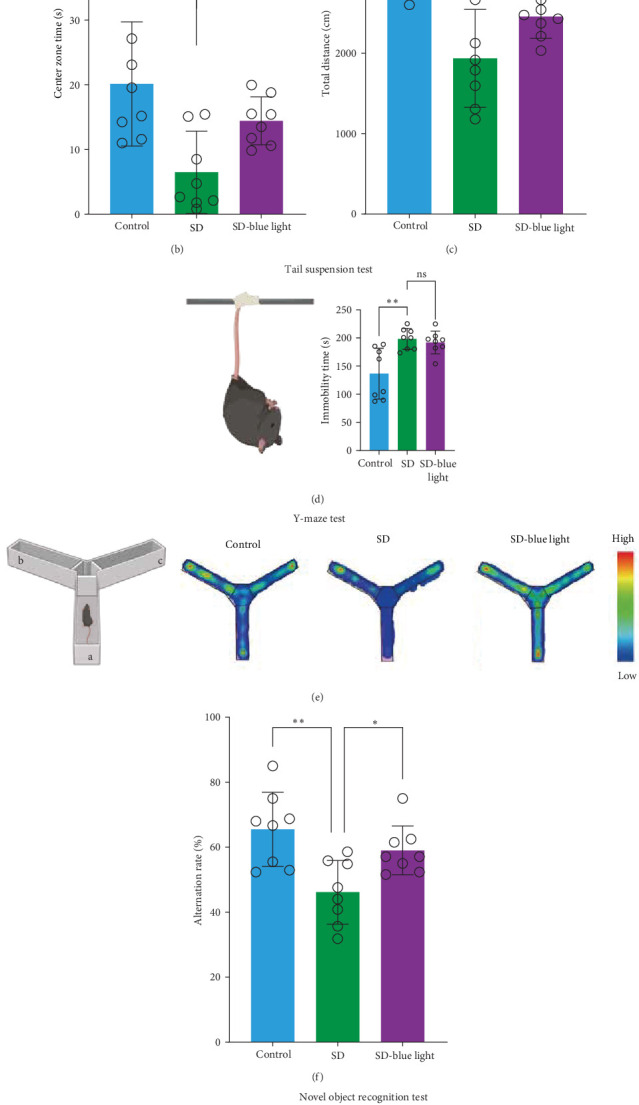
A 480 nm blue light exposure alleviates anxiety behaviors and improved learning and cognitive ability in SD mice. Typical route map (a), center zone time (b), and total distance (c) of each group in the open field test. (d) Schematic of tail suspension test (left) and immobility time (right). (e) Schematic of Y-maze (left) and typical examples of mice route map in the Y-maze apparatus. (f) % of spontaneous alternation in the Y-maze. (g) Schematic (left) and % of exploration rate (right) of novel object recognition experiment (two-tailed student's tests; *⁣*^*∗*^: *p* < 0.05; *⁣*^*∗∗*^: *p* < 0.01; *⁣*^*∗∗∗∗*^: *p* < 0.0001).

To assess the effects of 480 nm blue light exposure on SD mice, we conducted a series of behavioral tests. The OFT and TST were used to assess anxiety-like and depressive behaviors, respectively, while the NOR and Y-maze tests were conducted to evaluate spatial learning and memory abilities. Compared to SD mice, 480 nm blue light-exposed mice spent significantly more time in the center zone, had more center area entries, and exhibited increased time in the outer zone, with results comparable to those of the control group (*p* < 0.05, Table S7; Figure 7a,b and Figure S2). In addition, the total distance traveled was greater in the SD- blue light group compared to the SD group (Figure 7c), suggesting enhanced exploratory behavior. However, no statistically significant differences in immobility time were observed during the TST (Figure 7d and Table S7), indicating that blue light exposure had limited effects on depressive-like behaviors. 480 nm blue light exposure effectively alleviated anxiety-like behavior in SD mice. SD is known to impair spatial memory and cognitive function. Analysis of the spatial memory domain showed that mice exposed to 480 nm blue light had a significantly higher alternation rate in the Y-maze compared to the SD group (*p* < 0.05), with no significant differences from the control group (Figure 7e,f and Table S7). Furthermore, in the NOR test, mice in the SD-blue light group spent significantly more time exploring the novel object compared to SD mice, indicating improved recognition memory (Figure 7g and Table S7). These findings suggest that 480 nm blue light exposure alleviates anxiety-like behavior and mitigates deficits in cognitive function induced by SD in mice.

## 4. Discussion

Circadian rhythm disturbances and sleep disturbances are becoming increasingly prevalent in modern life, primarily due to exposure to multiple variables, including irregular light patterns, unconventional sleep schedules, and factors such as diet and individual susceptibility [28, 29]. This study investigates the effects of ipRGCs sensitive 480 nm blue light on the circadian rhythm of acutely SD mice, elucidating its mechanisms in regulating circadian rhythms and activating NSCs. Our findings reveal that exposure to 480 nm blue light significantly improved circadian rhythm disruption, cognitive impairment, and anxiety-like behaviors in SD mice, with notable differences in neural activation and expression of NSC stemness genes.

Existing evidence indicates that blue light exposure can exacerbate sleep disturbances and disrupt circadian rhythms, likely due to exposure to a specific, commonly encountered wavelength of light [8, 30]. In our study, after SD, mice exhibited a loss of rhythmic expression in the Cry2 gene, while the rhythmicity of Bmal1, Clock, and Per2 gene expression was partially preserved. Importantly, mice exposed to 480 nm blue light maintained the circadian rhythmicity of clock gene expression, accompanied by a noticeable phase advance. Additionally, we identified the intracellular cfos activation rhythm in the SCN and observed distinct spatiotemporal patterns of rhythm between the SD-blue light and SD groups, with the SD-blue light group showing activation levels similar to those of the control group. These findings provide visual evidence that blue light ameliorates SD-related disruptions in circadian rhythmicity. In a study simulating jet lag using wild-type male Wistar rats (6 h per week, 4 weeks), a normal phase but reduced amplitude and median of Per1 in the SCN were observed [31]. Additionally (E-box, CACGTG) of target genes such as Cry and Per is crucial for initiating transcription. Studies have shown that in genetic animal models, amplitudes and medians of SCN Per1, Per2, or cfos expression are congenitally reduced, with mutant mice and rats exhibiting faster phase shifts and entrainment into new light patterns compared to normal ones [32]. Therefore, by activating SCN neurons during the day–night cycle, we propose that optical manipulation altered the spontaneous motor activity and molecular circadian rhythms of the central SCN [33]. This suggests that blue light may activate SCN neuronal activity and upregulate cfos expression.

Intermittent tissue sampling of the SCN has reported intracellular rhythms related to NSCs in the central nervous system [13, 34]. However, precise spatiotemporal information regarding the rhythms of Nestin and Sox2 remains insufficient. We next examined the diurnal expression of Nestin and Sox2, observing fluctuating gene expression in SD mice, which suggests that SD may have a positive impact on NSCs function. A study detected that in adult mice, a significant proportion of Clock-positive cells overlapped with the neural progenitor cell marker Nestin and the neural stem cell marker glial fibrillary acidic protein, further underscoring the close relationship between clock genes and NSCs stemness genes [15]. Given the crucial role of stem cells in of mice, presenting a phase advance, which may be related to the effect tissue repair and regeneration of blue light, understanding the long-term effects of sleep and circadian rhythm disturbances on health is of significant importance. This change might be a response to stress in the SD group, where the body's regulatory mechanisms, disrupted by circadian rhythm abnormalities, attempt to protect neurons by upregulating stem gene expression [35]. Notably, the blue light-exposed group did not exhibit this fluctuation. Research indicates that deficiencies in these genes can lead to alterations in the transcriptome, resulting in reduced expression of core clock genes and neuropeptide receptor systems [36].

Next, we analyzed the rhythmic spontaneous motor activity of mice under an LD 12:12 environment. Despite the loss of circadian rhythm in spontaneous activity following SD, characterized by increased activity after lights off and decreased activity after lights on both the SD group and the SD-blue light group exhibited comparable motor abilities. This suggests that the impact of acute circadian disruption on normal motor function is negligible. Notably, the SD-blue light group exposed to 480 nm blue light showed a significant increase in both the frequency and intensity of activity during the dark period compared to the SD group, with activity levels resembling those of the control group (*p* < 0.05). This group also exhibited a smaller and delayed phase angle, consistent with findings from previous studies [30]. These results suggest that blue light may help preserve the integrity of the molecular clock within the SCN.

Sleep and circadian rhythm disruptions are known to severely impair cognitive and learning abilities, as well as mood, which we assessed using the Y-maze, NOR, OFT, and TST [37, 38]. In this study, SD led to reduced activity and exploratory behavior in the OFT, while the TST did not reveal significant signs of despair. This suggests that SD may primarily affect anxiety and overall activity rather than despair-like behavior. Importantly, the SD-blue light group demonstrated superior performance in spatial cognition and learning tasks compared to the SD group. Specifically, the SD-blue light group exhibited higher success rate in the Y-maze alternation tasks and better novel object recognition. Previous studies have shown that blue light can enhance working memory performance, and exposure to blue-enriched white light in the evening has been linked to improvements in cognitive function, alertness, and attention [39]. Furthermore, exposure to an inconsistent light pattern can lead to depressive-like behavior in mice, indicating that blue light exposure may improve anxiety-related behaviors, particularly since circadian rhythm disruptions can adversely affect mood and cognition [40]. In this study, we observed that blue light improved mood and cognitive function in mice, consistent with previous findings [41].

Our results indicate that exposure to 480 nm blue light significantly reduces the negative impact of circadian rhythm disruption on cognition and mood. However, it should be noted that this study has some limitations, such as the need for further exploration of the specific mechanisms underlying the effects of blue light, which will be addressed in future research. How blue light and stem cells influence the gene expression and stability of circadian rhythms is a theoretical hypothesis that requires further investigation and validation. In this study, we utilized an intensity of ~1300 lux of 480 nm blue light during the transition between day and night rhythms, while maintaining consistent lighting conditions for mice subjected to acute SD. This approach was designed to elucidate the mechanisms of action regarding circadian regularity, neural activation, and the impact on depressive conditions.

## 5. Conclusion

This study demonstrates that exposure to 480 nm blue light significantly improved circadian rhythm disruption, stabilized the expression of NSCs stemness genes, and mitigated associated emotional and cognitive function abnormalities induced by SD. These findings provide valuable insights into the interactions between blue light and the biological clock, paving the way for future research in this area.

## Data Availability

The data that support the findings of this study are available from the corresponding author upon reasonable request.

## Nomenclature

SD: Sleep deprivation
ipRGCs: Intrinsically photosensitive retinal ganglion cells
NSCs: Neural stem cells
SCN: Suprachiasmatic nucleus
ZTs: Zeitgebers
TBI: Traumatic brain injury
LD: Light–dark
OFT: Open field test
TST: Tail suspension test
NOR: Novel object recognition test.

## Supporting Information

Supporting Information 1 Figure S1. Clock genes mRNA expression and the rhythmic changes of the fitted cosine function and relative expression changes at different irradiation intensities. mRNA expression of Bmal1 (a), Clock (b), Cry2 (c) and Per2 (d). Figure S2. Number of enter to center zone (a) and outer zone time rate (b) of each group in the open field test. Figure S3. Activated cfos protein of the SCN slice. Table S1. PCR Primer Sequence. Table S2. Parameters of central clock genes expression of Bmal1, Clock, Per2 mRNA in fitting cosine function. Table S3. Parameters of peripheral (liver) clock genes expression of Bmal1, Clock, Per2 mRNA in fitting cosine function.

Supporting Information 2 Table S4. Summary statistics of t-test and ANOVA for central clock genes mRNA expression. Table S5. Summary statistics of t-test and ANOVA for peripheral (liver) clock genes mRNA expression. Table S6. Summary statistics of t-test and ANOVA for immunofluorescence of cfos, Nestin, and Sox2. Table S7. Summary statistics of t-test and ANOVA for behaviors test.
