# Supplementary material for: ipRGCs Sensitive Blue Light Exposure Promotes the Robustness of Circadian and Neural Stem Cells in Sleep Deprived Conditions
Source: Stem Cells Int. 2025 Jul 24;2025:8828183. doi: 10.1155/sci/8828183 (PMC12313382; doi:10.1155/sci/8828183)
Supplement: Supporting Information 1 — Figure S1. Clock genes mRNA expression and the rhythmic changes of the fitted cosine function and relative expression changes at different irradiation intensities. mRNA expression of Bmal1 (a), Clock (b), Cry2 (c) and Per2 (d). Figure S2. Number of enter to center zone (a) and outer zone time rate (b) of each group in the open field test. Figure S3. Activated cfos protein of the SCN slice. Table S1. PCR Primer Sequence. Table S2. Parameters of central clock genes expression of Bmal1, Clock, Per2 mRNA in fitting cosine function. Table S3. Parameters of peripheral (liver) clock genes expression of Bmal1, Clock, Per2 mRNA in fitting cosine function. [file 8828183.f1.docx]

**ipRGCs** **Sensitive** **Blue Light Exposure Promotes the** **Robustness of Circadian and Neural Stem Cells in Sleep Deprived Conditions**

Table S1. PCR Primer Sequence.

| Primer Name | Forward primer | Reverse primer |
| --- | --- | --- |
| b-Actin | 5'-GAAGATCAAGATCATTGCTCC-3' | 5'-TACTCCTGCTTGCTGATCCA-3' |
| Bmal1 | 5'-TGCCACCAATCCATACAC-3' | 5'-TGCCACCAATCCATACAC-3' |
| Clock | 5'-TGCTGGAAAGTGACTCCTTAACCC-3' | 5'-TGCTGGAAAGTGACTCCTTAACCC-3' |
| Cry2 | 5'-CAAAGGACTACGGCTCCACG-3' | 5'-TGCAGTAGGAACCTCCATCG-3' |
| Per2 | 5'-CGCGGCGAAGCGCTTATT-3' | 5'-GTGGGACTGGTGGGACTTG-3' |
| Sox2 | 5'-AGGAAAGGGTTCTTGCTGGG-3' | 5'-ACGAAAACGGTCTTGCCAGT-3' |
| Nestin | 5'-CGGGAGAGTCGCTTAGAGGT-3' | 5'-TTCCACAGCCAGCTGGAAC-3' |


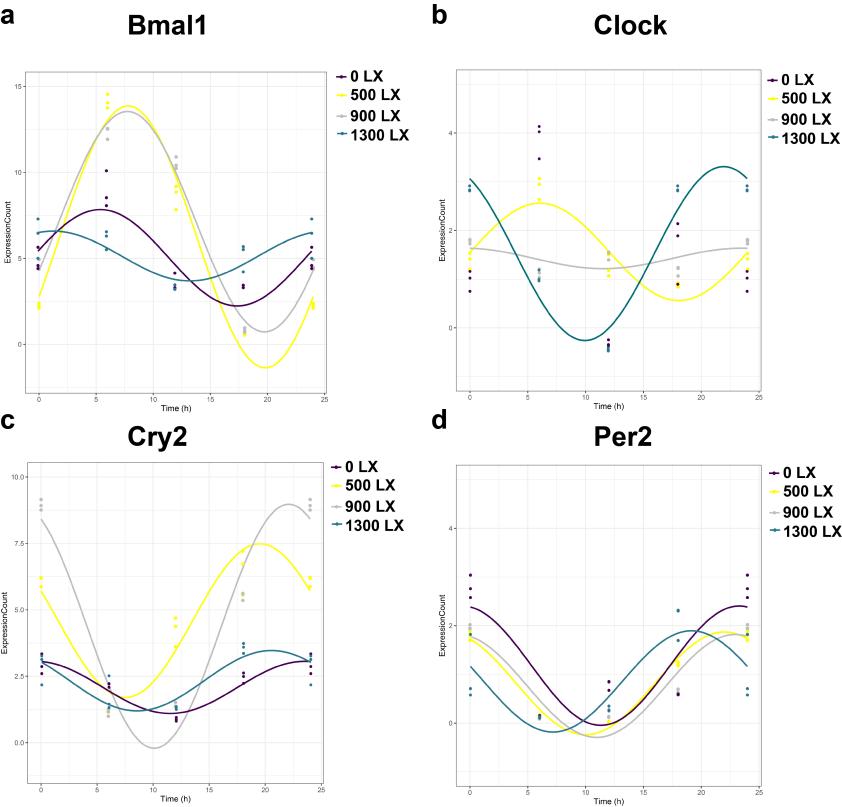


Figure S1. Clock genes mRNA expression and the rhythmic changes of the fitted cosine function and relative expression changes at different irradiation intensities. mRNA expression of Bmal1 (a), Clock (b), Cry2 (c) and Per2 (d).

Table S2. Parameters of central clock genes expression of Bmal1, Clock，Cry2，Per2 mRNA in fitting cosine function.

| Genes | Groups | Mesor | Amplitude | Phase_radians | Peak_time_hours |
| --- | --- | --- | --- | --- | --- |
| Bmal1 | Control | 1.352 | 0.747 | 3.974 | 15.178 |
|  | SD | 2.618 | 2.227 | 2.921 | 11.157 |
|  | SD-blue light | 2.435 | 1.41 | 2.253 | 8.606 |
| Clock | Control | 4.015 | 4.683 | 5.958 | 22.759 |
|  | SD | 5.857 | 4.244 | 2.696 | 10.299 |
|  | SD-blue light | 8.473 | 9.159 | 1.387 | 5.296 |
| Cry2 | Control | 1.634 | 1.433 | 6.281 | 23.993 |
|  | SD | 2.113 | 1.097 | 1.532 | 5.853 |
|  | SD-blue light | 2.11 | 0.871 | 1.237 | 4.724 |
| Per2 | Control | 0.641 | 0.568 | 0.514 | 1.961 |
|  | SD | 0.944 | 1.036 | 1.569 | 5.995 |
|  | SD-blue light | 0.623 | 0.671 | 0.613 | 2.341 |

Table S3. Parameters of peripheral (liver) clock genes expression of Bmal1, Clock，Cry2，Per2 mRNA in fitting cosine function.

| Genes | Groups | Mesor | Amplitude | Phase_radians | Peak_time_hours |
| --- | --- | --- | --- | --- | --- |
| Bmall | Control | 3.539 | 2.409 | 3.467 | 13.244 |
|  | SD | 1.769 | 1.608 | 2.501 | 9.552 |
|  | SD-blue light | 3.052 | 1.315 | 2.49 | 9.509 |
| Clock | Control | 0.553 | 0.364 | 6.098 | 23.294 |
|  | SD | 0.617 | 0.334 | 5.777 | 22.068 |
|  | SD-blue light | 0.388 | 0.164 | 5.322 | 20.329 |
| Cry2 | Control | 1.253 | 0.181 | 3.535 | 13.501 |
|  | SD | 1.897 | 0.526 | 3.963 | 15.139 |
|  | SD-blue light | 2.544 | 2.369 | 5.119 | 19.555 |
| Per2 | Control | 0.379 | 0.502 | 0.251 | 0.958 |
|  | SD | 0.386 | 0.532 | 0.009 | 0.035 |
|  | SD-blue light | 0.22 | 0.234 | 5.058 | 19.322 |


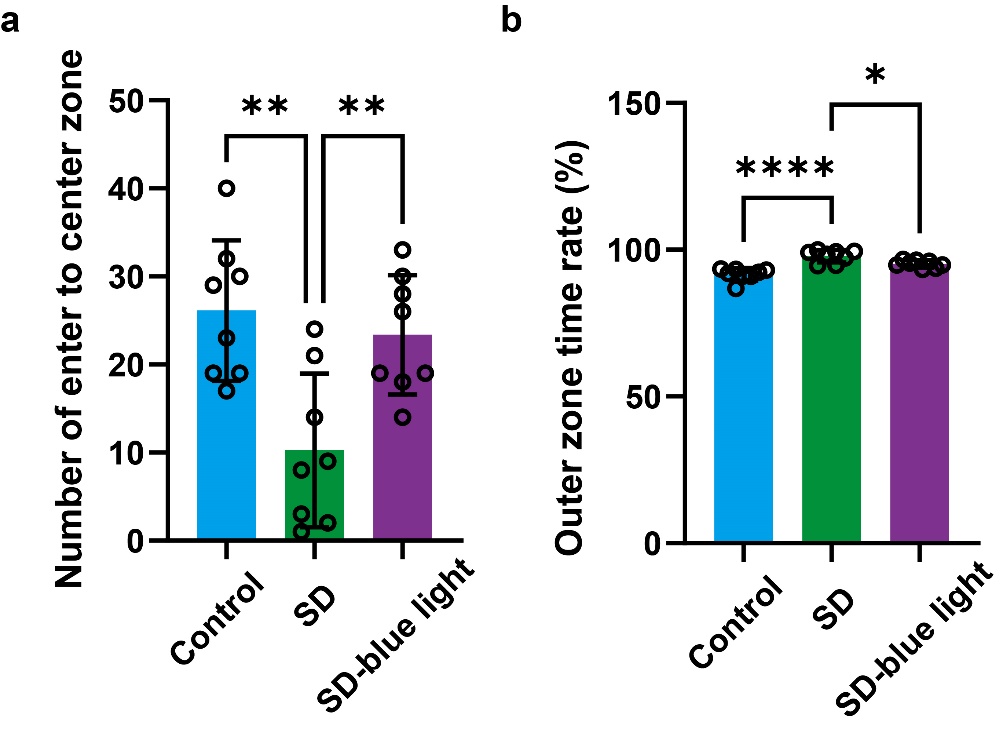


Figure S2. Number of enter to center zone (a) and outer zone time rate (b) of each group in the open field test. SD= sleep deprivation.


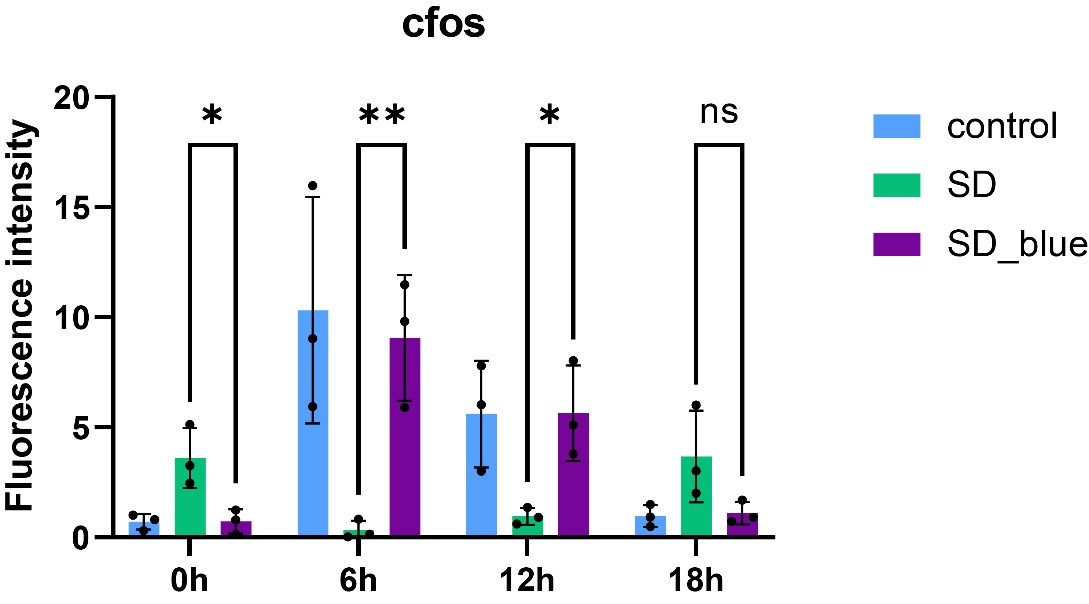


Figure S3. Activated cfos protein of the SCN slice.
